# Effects of climatic factors on COVID-19 transmission in Ethiopia

**DOI:** 10.1038/s41598-022-24024-9

**Published:** 2022-11-16

**Authors:** Fitsum Bekele Endeshaw, Fentabil Getnet, Awoke Misganaw Temesgen, Alemnesh H. Mirkuzie, Latera Tesfaye Olana, Kefyalew Addis Alene, Solomon Kibret Birhanie

**Affiliations:** 1 National Data Management Center for Health, Ethiopian Public Health Institute, Addis Ababa, Ethiopia; 2 School of Public Health, Faculty of Health Sciences, Curtin University, Kent St, Bentley, WA 6102 Australia; 3 Program in Public Health, University of California, Irvine, CA USA; 4 Geospatial and Tuberculosis Research Team, Telethon Kids Institute, Nedlands, Australia

**Keywords:** Viral infection, Risk factors

## Abstract

Climatic conditions play a key role in the transmission and pathophysiology of respiratory tract infections, either directly or indirectly. However, their impact on the COVID-19 pandemic propagation is yet to be studied. This study aimed to evaluate the effects of climatic factors such as temperature, rainfall, relative humidity, sunshine duration, and wind speed on the number of daily COVID-19 cases in Addis Ababa, Ethiopia. Data on confirmed COVID-19 cases were obtained from the National Data Management Center at the Ethiopian Public Health Institute for the period 10th March 2020 to 31st October 2021. Data for climatic factors were obtained from the Ethiopia National Meteorology Agency. The correlation between daily confirmed COVID-19 cases and climatic factors was measured using the Spearman rank correlation test. The log-link negative binomial regression model was used to fit the effect of climatic factors on COVID-19 transmission, from lag 0 to lag 14 days. During the study period, a total of 245,101 COVID-19 cases were recorded in Addis Ababa, with a median of 337 new cases per day and a maximum of 1903 instances per day. A significant correlation between COVID-19 cases and humidity was observed with a 1% increase in relative humidity associated with a 1.1% [IRRs (95%CI) 0.989, 95% (0.97–0.99)] and 1.2% [IRRs (95%CI) 0.988, (0.97–0.99)] decrease in COVID-19 cases for 4 and 5 lag days prior to detection, respectively. The highest increase in the effect of wind speed and rainfall on COVID-19 was observed at 14 lag days prior to detection with IRRs of 1.85 (95%CI 1.26–2.74) and 1.078 (95%CI 1.04–1.12), respectively. The lowest IRR was 1.109 (95%CI 0.93–1.31) and 1.007 (95%CI 0.99–1.02) both in lag 0, respectively. The findings revealed that none of the climatic variables influenced the number of COVID-19 cases on the day of case detection (lag 0), and that daily average temperature and sunshine duration were not significantly linked with COVID-19 risk across the full lag period (*p* > 0.05). Climatic factors such as humidity, rainfall, and wind speed influence the transmission of COVID-19 in Addis Ababa, Ethiopia. COVID-19 cases have shown seasonal variations with the highest number of cases reported during the rainy season and the lowest number of cases reported during the dry season. These findings suggest the need to design strategies for the prevention and control of COVID-19 before the rainy seasons.

## Introduction

Since Corona Virus Disease 2019 (COVID-19) was officially declared by the World Health Organization (WHO) on 11 March 2020 as a global pandemic, the number of deaths and daily confirmed new cases have increased in every corner of the world. The pandemic is a serious global public health crisis affecting the physical, mental, social, and economic well-being of human beings^1–3^. Following the first reported cases of COVID-19 in Wuhan, China in late December 2019, the virus has quickly spread across the world^4^. As of 11 March 2022, there were more than 453 million confirmed COVID-19 cases and over 6 million associated deaths around the globe^3^. As of 11 March 2022, COVID-19 has infected 466,064 persons in Ethiopia, with over two-thirds of the COVID-19 patients (310,402, 66.6%) reported from the capital city, Addis Ababa^5^.

Climatic conditions play a key role in the transmission and pathophysiology of respiratory tract infections, either directly or indirectly. Studies showed that climatic factors such as humidity, rainfall, temperature, UV intensity, wind speed and air pollutants responsible for the survival, viability, and transmission of infectious viruses^6,7^. However, the impacts of these climatic factors on COVID-19 transmission are yet to be studied. While few studies investigated the effects of climatic factors on the COVID-19 pandemic, they have reported mixed findings. Some studies reported that climatic factors have been shown to impact the viability, droplet nuclei and fomite transmission of COVID-19^8–10^. A study conducted in sixteen African countries showed both mean temperature and humidity have an overall inverse relationship with daily confirmed COVID-19 cases^11^. The African study showed a 1 °C increase in temperature and a 1% increase in humidity to be associated with a 15.1% and 3.6% reduction in the number of daily confirmed COVID-19 cases, respectively^11^. Similarly, studies conducted in India, USA and Mexico showed that temperature has a positive relationship with the number of daily confirmed COVID-19 cases^9,12^. In contrast, studies conducted in Brazil, Peru, Ghana and South Africa showed an inverse relationship between the daily confirmed number of COVID-19 cases and temperature, rainfall and humidity^13,14^. This highlights that the effect of climate factors on COVID-19 transmission varies across locations and climatic zones^15–17^. This inconsistent relationship between the reported number of COVID-19 cases and climatic factors calls for further investigation through the application of robust and repeatable methods.

Investigating the potential effects of climatic factors including temperature, rainfall, relative humidity, sunshine duration, and wind speed on the transmission of COVID-19 is crucial to support context-specific decisions on public health measures. Therefore, this study was aimed at predicting the effect of climatic factors, on the spread of the COVID-19 pandemic in Addis Ababa, Ethiopia.

## Data and methods

### Study area

**Figure 1:**
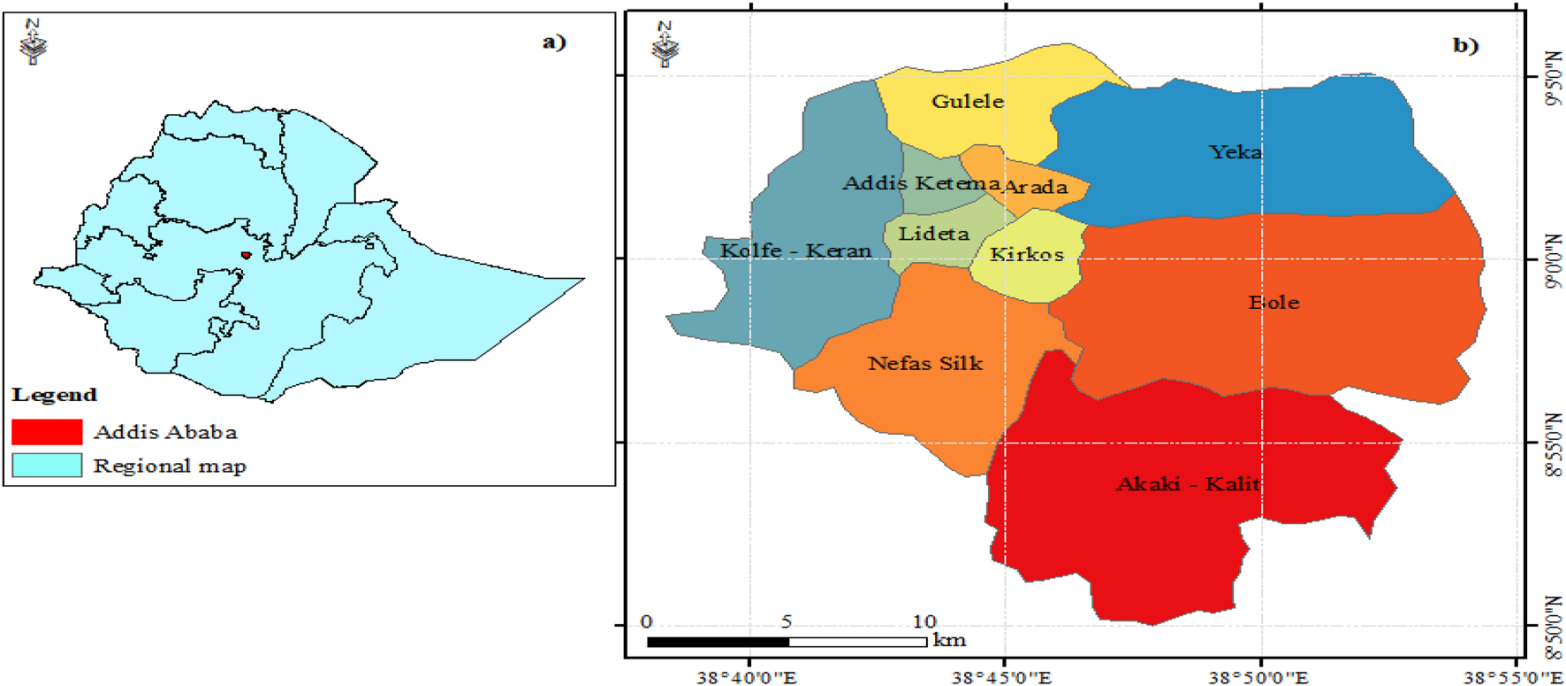
Map showing the location of the study area: (**a**) regional map of Ethiopia, (**b**) Addis Ababa sub-city.

This study was conducted in the capital city of Ethiopia, Addis Ababa, which is located between 8.84°–9.1° N and 38.65°–38.9° E. The area experiences a bimodal rainfall characteristic, with an annual average rainfall of 61.1 mm between October and January and 834.9 mm between June and September^18^. The lowest temperature occurs between October and January with an average of 7.8 °C and the highest occurs between February and May with an average of 25.3 °C. The 2020 projected population of Addis Ababa is 3,686,001^19^. As of October 2021, the total number of confirmed COVID-19 cases in Addis Ababa was 245,247, which is double the total 119,509 COVID-19 cases reported across the 11 administrative regions in the country (Fig. 1).

### Data sources

#### COVID-19 incidence data

The Ethiopian Public Health Institute's National Data Management Center for Health provided information on the number of daily confirmed COVID-19 cases in Addis Ababa. The study includes all new COVID-19 cases reported to the surveillance system between May 10, 2020, and October 31, 2021. For this time series study, 540 (N) facilities from governmental and non-governmental health facilities in Addis Ababa were used to aggregate daily case counts of positive COVID-19 diagnoses. Because all cases were imported and in isolation facilities at airports, entry ports, and quarantine sites, data from 13 March to 9 May 2020 were excluded from this investigation. Community transmission was officially reported by the government of Ethiopia on 10 May 2020.

#### Climate data

Climatic data were obtained from the Ethiopia Meteorology Institute database collected between 26 April 2020 and 31 October 2021. The Institute records climate data on daily basis in synoptic and first-class meteorological stations located at two sites in Addis Ababa. The climatic data included rainfall (mm), minimum and maximum temperature (°C), relative humidity (%), sunshine duration (h) and wind speed (m/s). Since climate factors have a lag cumulative effect on infectious disease transmission, a COVID-19 incubation period of 1–14 days was taken into account in the climatic parameters and data were collected accordingly.

### Statistical analysis

Descriptive statistics including mean, median, standard deviations, percentiles, minimum and maximum were used to summarize exposure variables such as rainfall (mm), minimum and maximum temperature (°C), relative humidity (%), sunshine duration (h) and wind speed (m/s) as well as dependent variables (i.e., COVID-19 daily confirmed cases). Data normality for linearity assumptions of the response variable was not fulfilled (Shapiro–Wilk test of normality, *p* ≤ 0.0001). Logarithmic, inverse/reciprocal, Box-Cox, square root, and exponential techniques failed to meet the required assumptions, hence non-parametric correlation estimate (Spearman correlation coefficient) was applied. The Spearman correlation coefficient was used to estimate the relationship between rainfall (mm), minimum and maximum temperature (°C), relative humidity (%), sunshine duration (h) and wind speed (m/s) with daily confirmed COVID-19 cases, and a bivariate, two-tailed analysis with 95% confidence intervals was applied. Since the number of COVID-19 cases was a count variable, we assumed that it followed a Poisson distribution. However, the count data on the daily confirmed cases was over-dispersed and the mean value was smaller than their variance. Thus, instead of Poisson regression, a negative binomial regression model was employed to quantify the relationship between climatic valuables and COVID-19 cases.

Considering the suggested latent period for COVID-19 which is 1–14 days long^20^, a moving-average concept to compensate for the potential lag time effects of climatic factors on daily confirmed cases was applied^21^. Consequently, 14 days moving average for rainfall (mm), mean temperature (°C), relative humidity (%), sunshine duration (h) and wind speed (m/s) were used. Before fitting the model, all covariates were checked for multi-collinearity using a Pearson correlation coefficient and those variables showing multicollinearity were excluded from the final model.

The negative binomial regression model was fitted as follows:

$${\log}\mu t={\beta }_{0}+{\beta }_{1}{T}_{mean}+{\beta }_{2}{R}_{h}+{\beta }_{3}Rain+{\beta }_{4}wind+{\beta }_{5}sunshine \; duration+error\;  term$$

where μ_t_ is an expected confirmed case at day t; β_0_ represents the intercept, β_1_, β_2_, β_3_, β_4 and_ β_5_ are the regression coefficients of 1–14 days moving average of the daily mean temperature (T_mean_), relative humidity (Rh), rainfall, wind, and sunshine duration, respectively. Data processing and analysis were done using R software version 4.1.1^22^.

Further, daily variations of mean temperature, rainfall, relative humidity, wind speed and sunshine duration were graphically inspected along with a comparison of the daily variation in COVID-19 cases during the study period.

### Ethical approval

The authors declare that all data used in the present study were accessed from the repository hub of the National Data Management Center for Health at the Ethiopian Public Health Institute, where the author currently works. As the authors used publicly available data, ethical approval was not required.

## Results

Between May 10, 2020, and October 31, 2021, a total of 245,101 COVID-19 cases were registered in Addis Ababa. The largest number of cases reported per day was 1,903, and the median number of new cases per day was 337 (Table 1).

**Table 1:** Summary statistics of daily confirmed Covid-19 new cases and climate variables in Addis Ababa (N = 540).

Variables	Minimum	P(25)^a^	Median	Mean	SD^b^	P(75)	Maximum	Sum
Daily new cases (person)	0	171.3	337	454	390.8	628.8	1903	245,101
Rainfall (mm)	0	0	0	4.3	7.5	5.7	43.8	
Relative humidity (%)	9.5	37	52.8	52.5	18.7	67.4	95	
Minimum temperature (°C)	5.2	10.4	11.7	11.3	1.8	12.6	16.3	
Maximum temperature (°C)	14.4	22.1	24	23.7	2.5	25.5	29.3	
Average temperature (°C)	13	16.5	17.4	17.5	1.4	18.4	21.2	
Wind (m/s)	0.1	0.7	1	1.1	0.5	1.4	2.9	
Sunshine duration (hours)	0	3.5	6.6	6.3	3.4	9.9	11	

Data source: from 10th May 2020 to 31st October 2021.

^a^Percentile and ^b^Standard deviation.

The lowest and highest temperatures recorded throughout the research period were 5.2 °C and 29.3 °C, respectively, with a median of 11.7 °C and 29.3 °C. The minimum and maximum average daily temperature ranged from 13 to 21.2 °C with a median of 17.5 °C. The highest relative humidity and rainfall values were 95% and 43.8 mm, respectively, with a median of 52.8% and 0 mm. During the study period, the greatest wind speed and sunshine duration were 2.9 m/s and 11 h, respectively, with a median of 1.1 m/s and 6.6 h.

The minimum sunshine duration ranged from 0 h during the main rainy season to 11 h during the dry season with a standard deviation of 3.4 (Table 1).

### Correlation analysis between climate factors and COVID-19 positive cases

Rainfall, relative humidity, and minimum temperature have a significant and inverse relationship with cases of daily confirmed positive COVID-19, whereas maximum temperature, mean daily temperature, wind speed, and sunshine duration has a direct relationship with low magnitude, according to the Spearman correlation analysis (Table 2).

**Table 2:** Spearman’s rank correlation coefficient between daily confirmed positive COVID-19 cases and climate variables for Lag 0 to 14 days in Addis Ababa.

Lag time	Rainfall	Relative humidity	Tmin	Tmax	Tmean	Wind	Sunshine hours
0	− 0.082	− 0.121**	− 0.152**	0.096*	0.020	0.096*	0.101*
1	− 0.082	− 0.115**	− 0.138**	0.091*	0.024	0.103*	0.085*
2	− 0.080	− 0.115**	− 0.159**	0.088*	0.018	0.106*	0.092*
3	− 0.069	− 0.110*	− 0.166**	0.082	0.004	0.115**	0.097*
4	− 0.060	− 0.110*	− 0.174**	0.077	− 0.007	0.123**	0.095*
5	− 0.056	− 0.107*	− 0.183**	0.074	− 0.016	0.130**	0.092*
6	− 0.049	− 0.100*	− 0.192**	0.066	− 0.027	0.127**	0.086*
7	− 0.049	− 0.101*	− 0.201**	0.062	− 0.036	0.125**	0.079
8	− 0.048	− 0.103*	− 0.212**	0.055	− 0.046	0.123**	0.081
9	− 0.045	− 0.102*	− 0.224**	0.047	− 0.058	0.121**	0.081
10	− 0.043	− 0.100*	0.040	0.040	− 0.071	0.122**	0.081
11	− 0.039	− 0.099*	− 0.240**	0.036	− 0.078	0.128**	0.081
12	− 0.039	− 0.097*	− 0.247**	0.032	− 0.084	0.130**	0.081
13	− 0.037	− 0.093*	− 0.253**	0.027	− 0.094*	0.133**	0.083
14	− 0.041	− 0.092*	− 0.262**	0.024	− 0.107*	0.133**	0.084

Tmin, Tmax and Tmean are minimum, maximum and mean temperature, respectively.

*Correlation is significant at the 0.05 level (2-tailed).

**Correlation is significant at the 0.01 level (2-tailed).

The correlation analysis revealed that minimum temperature (in °C) had an inverse significant correlation with an increased strength at lag 14 days prior to detection compared to the rest lag periods with the occurrence of daily new cases of COVID-19 (− 0.262, *p* < 0.01), conversely, the maximum temperature had a non-significant direct relationship with the occurrence of daily confirmed COVID-19 case except for lag 0 (day of detection) to lag 2 days.

The strength and statistical significance between minimum temperature (r_s_ = − 0.262, *p* < 0.01), mean temperature (r_s_ = − 0.107, *p* < 0.05), wind speed (r_s_ = 0.133, *p* < 0.01) and daily confirmed COVID-19 positive cases increased as cumulative lag period increased (Table 2). However, among the climatic factors only maximum temperature (up to 2 days lag period) and sunshine duration (up to 6 days lag) had a statistically significant relationship with daily confirmed COVID-19 positive cases in Addis Ababa. The lag periods between minimum temperature, relative humidity, wind, sunshine duration, and daily confirmed COVID-19 positive cases showed a weak statistically significant correlation for most of the lag periods at a 5% significance level. Conversely, rainfall, maximum and minimum temperature showed an absence of a statistically significant correlation with daily confirmed COVID-19 positive cases for most of the lag periods (Table 2).

### Effects of climate variables on daily confirmed positive COVID-19 cases

Table 3 shows the results of a log-link negative binomial regression model that indicates the associational effect between daily confirmed positive COVID-19 cases and the meteorological factors such as, mean daily temperature, rainfall, relative humidity, sunshine duration, and wind speed.

**Table 3:** Effects of climate factors on COVID-19 case counts in Addis Ababa.

Lag days	Rainfall			Relative humidity			Mean temperature			Wind			Sunshine		
	IRR	*p* value	95% CI	IRR	*p* value	95% CI	IRR	*p* value	95% CI	IRR	*p* value	95% CI	IRR	*p* value	95% CI
0	1.007	0.23	0.99–1.02	0.995	0.14	0.98–1.02	1.049	0.12	0.98–1.12	1.109	0.23	0.93–1.31	0.996	0.81	0.96–1.03
1	1.010	0.107	0.99–1.02	0.994	0.06	0.98–1	1.052	0.10	0.99–1.12	1.141	0.13	0.96–1.35	0.986	0.44	0.95–1.02
2	1.020	0.018	1.01–1.04	0.992	0.07	0.98–1.001	1.057	0.12	0.98–1.13	1.199	0.08	0.98–1.47	0.988	0.60	0.94–1.03
3	1.029	0.004	1.01–1.05	0.991	0.08	0.98–1.001	1.046	0.23	0.97–1.13	1.298	0.02	1.04–1.63	0.986	0.61	0.94–1.04
4	1.039	0.001	1.02–1.06	0.989	0.04	0.97–0.99	1.040	0.32	0.96–1.12	1.374	0.01	1.07–1.76	0.978	0.46	0.92–1.04
5	1.042	0.001	1.02–1.07	0.988	0.04	0.97–0.99	1.034	0.40	0.95–1.12	1.452	0.01	1.11–1.90	0.969	0.31	0.91–1.03
6	1.048	0.000	1.02–1.08	0.988	0.06	0.97–1.0	1.031	0.46	0.95–1.12	1.526	0.00	1.14–2.04	0.970	0.37	0.91–1.04
7	1.051	0.000	1.02–1.08	0.989	0.09	0.97–1.02	1.029	0.50	0.95–1.12	1.609	0.00	1.18–2.19	0.972	0.41	0.91–1.04
8	1.056	0.000	1.03–1.08	0.988	0.1	0.97–1.02	1.021	0.62	0.94–1.11	1.633	0.00	1.19–2.24	0.974	0.46	0.91–1.05
9	1.062	0.000	1.03–1.09	0.987	0.09	0.97–1.02	1.011	0.80	0.93–1.10	1.665	0.00	1.2–2.31	0.975	0.51	0.91–1.05
10	1.068	0.000	1.04–1.10	0.986	0.07	0.97–1.02	0.999	0.97	0.92–1.09	1.700	0.00	1.21–2.38	0.973	0.49	0.90–1.05
11	1.074	0.000	1.04–1.11	0.984	0.06	0.96–1.01	0.988	0.79	0.9–1.08	1.728	0.00	1.22–2.45	0.971	0.47	0.89–1.05
12	1.077	0.000	1.04–1.11	0.983	0.06	0.96–1.01	0.977	0.62	0.89–1.07	1.763	0.00	1.23–2.52	0.968	0.45	0.89–1.05
13	1.077	0.000	1.04–1.12	0.984	0.09	0.96–1.03	0.967	0.484	0.88–1.06	1.823	0.00	1.26–2.64	0.971	0.51	0.89–1.06
14	1.078	0.000	1.04–1.12	0.983	0.09	0.96–1.03	0.953	0.323	0.86–1.05	1.854	0.00	1.26–2.72	0.969	0.49	0.89–1.06

*IRR* Incidence rate ratio, *CI* confidence interval.

From the analysis of the log-link negative binomial regression model, among the five climate parameters, none of them had a statistically significant effect on daily confirmed COVID-19 cases on the day of detection (lag 0) or at 2 lag days prior to detection (i.e., when transmission probably occurred).

The results further indicate that among the climate factors included in the study, mean temperature and sunshine duration had no significant role in COVID-19 incidence for all lag days (*p* > 0.05), while relative humidity had a positive effect on increasing COVID-19 cases for 4 and 5 cumulative lag days prior to the detection of COVID-19 cases (*p* < 0.05). A 1% increase in relative humidity was associated with 1.1% and 1.2% decreased COVID-19 cases for 4 and 5 lag days prior to detection.

The magnitude of rainfall was significantly associated with the risk of daily COVID-19 cases for all lag days except for lag zero (day of detection) to 1 day (prior to detection of the virus), while an increased risk of daily COVID-19 cases was associated with the increased magnitude of wind speed for all lag periods except at the day of detection (lag 0) to 2 days.

The maximum increased effect of wind speed and rainfall on COVID-19 cases was observed at the 14-day lag prior to infection detection with respective IRRs being 1.854 (95%CI 1.26–2.74) and 1.078 (95%CI 1.04–1.12). The lowest IRRs were 1.109 (95%CI 0.93–1.31) for wind speed and 1.007 (95%CI 0.99–1.02) for rainfall, both in lag 0.

Overall, the negative binomial regression model analysis showed that with increased cumulative effects of rainfall and wind speed (prior to detection of COVID-19 cases), the number of positive COVID-19 cases significantly increased over the study area when considering other factors constant. This suggests that rainfall and wind speed had a great role in the transmission of the COVID-19 outbreak (Table 3).

### Seasonal and daily variation of climate factors against daily confirmed new cases

**Figure 2:**
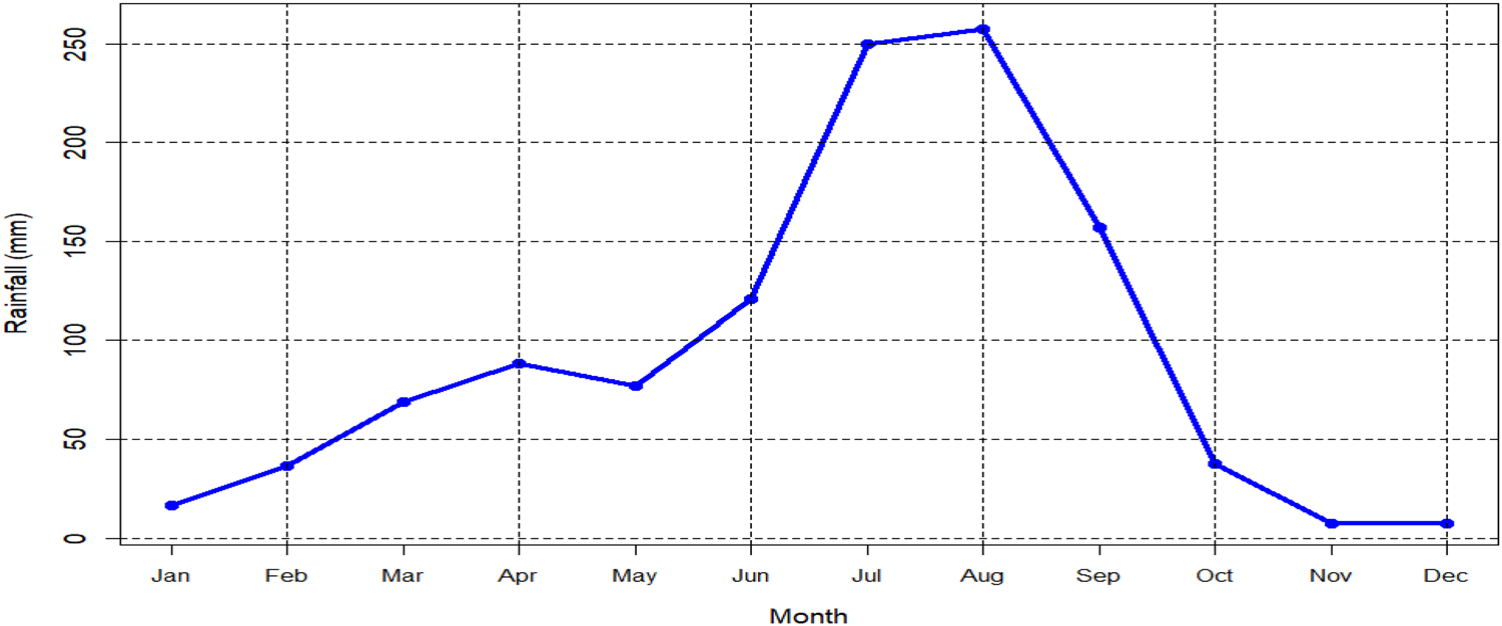
Rainfall climatology (1981–2010) of Addis Ababa.

**Figure 3:**
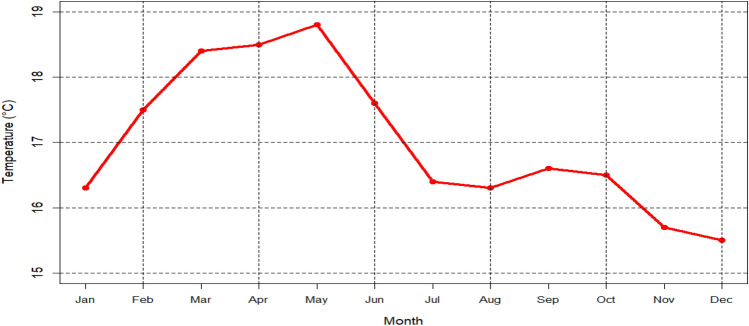
Mean temperature climatology (1981–2010) of Addis Ababa.

As can be seen from Fig. 2, on average, the highest and lowest rainfall record in Addis Ababa were from June to September and October to November, respectively. However, the maximum (May) and minimum (December) temperatures recorded in Addis Ababa were during the second small rainy season (February to May) and October to January period (Fig. 3).

**Figure 4:**
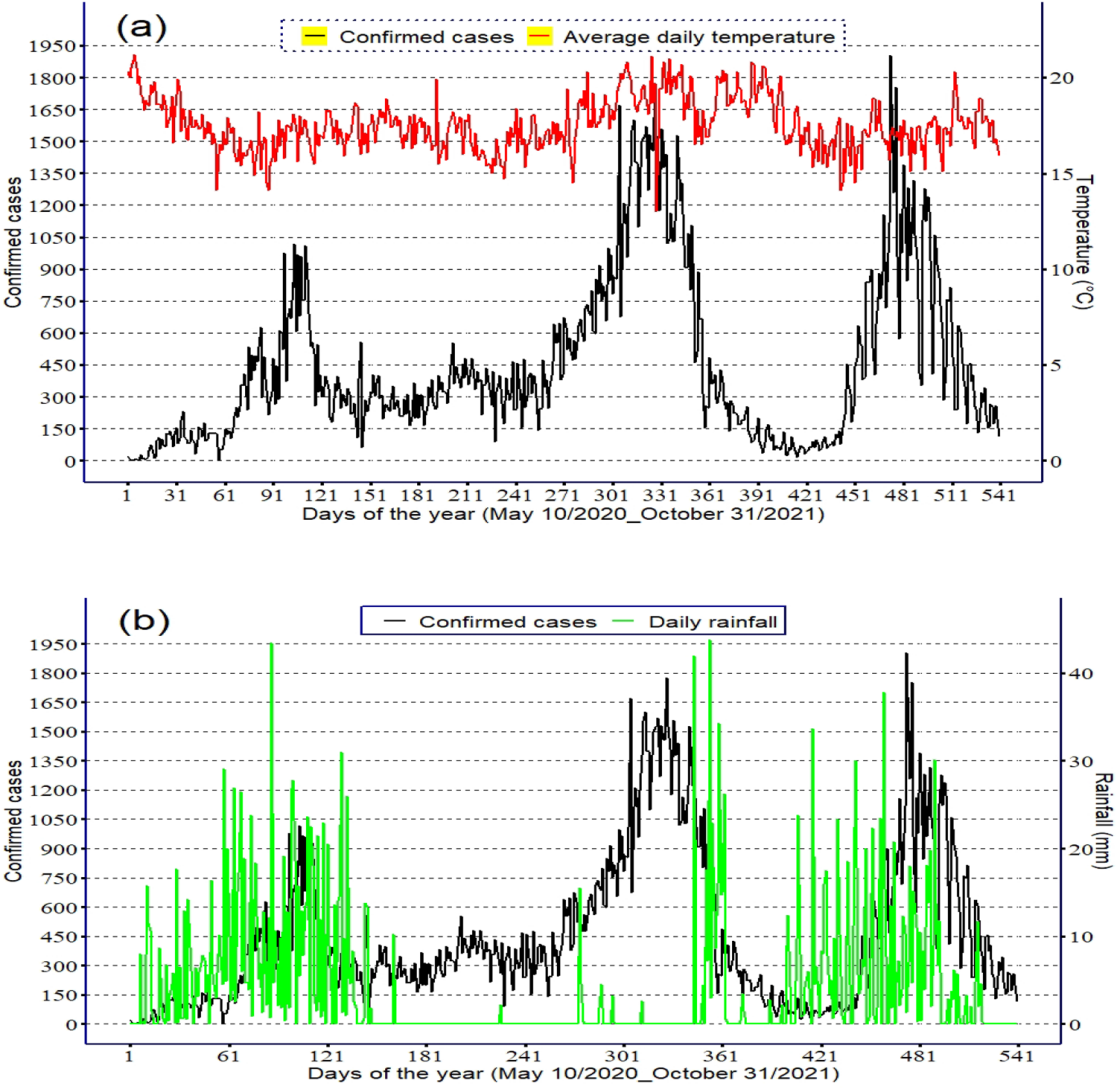

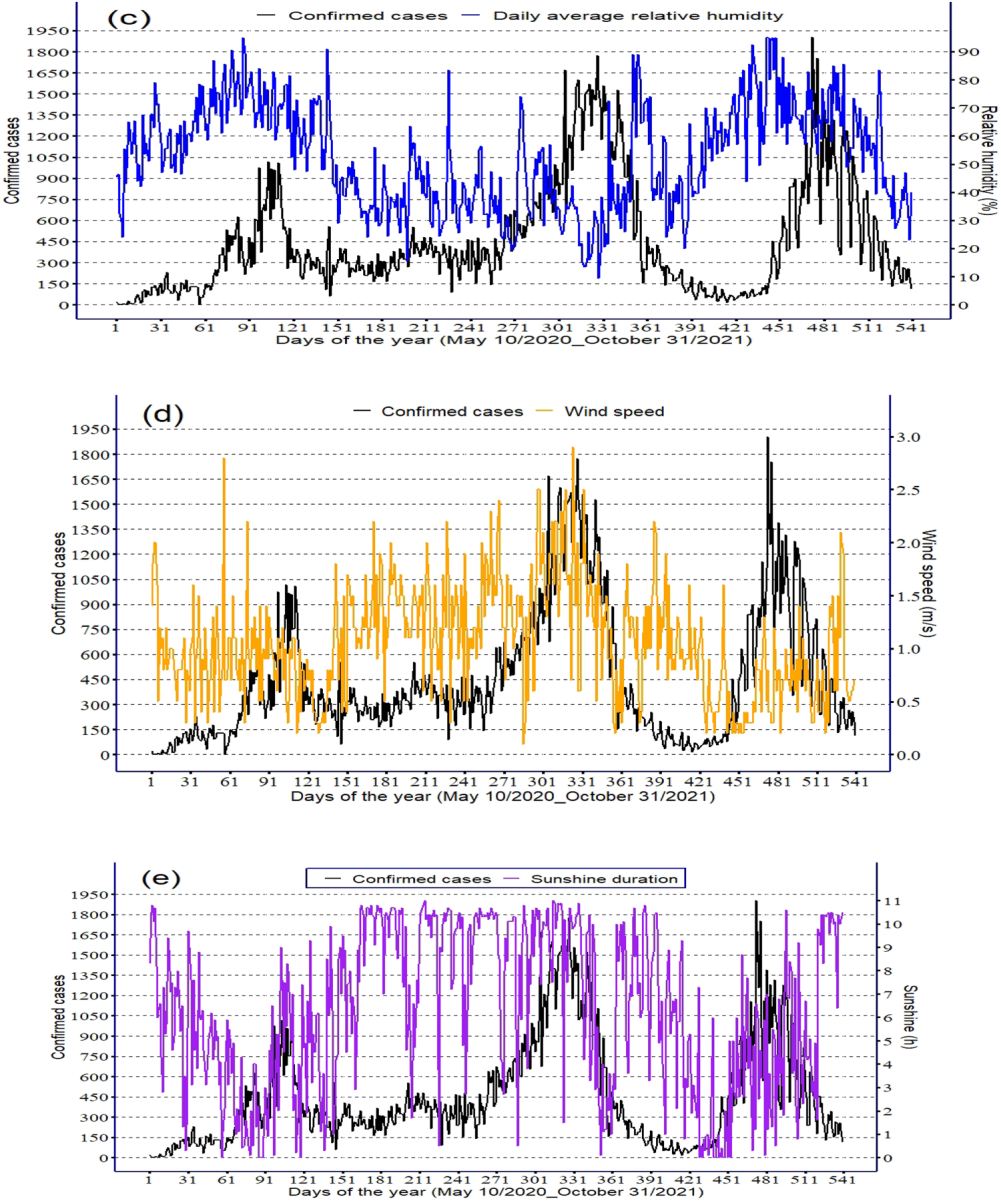
Daily variation of sunshine duration and (**a**) Average daily temperature, (**b**) Rainfall, (**c**) Relative humidity, (**d**) Wind speed and (**e**) Sunshine duration from 10 May 2020 to 31 October 2021 in Addis Ababa.

As Fig. 4a illustrates, Addis Ababa experienced 3 COVID-19 peak periods since community-level transmission occurred. The two highest numbers of COVID-19 cases per day were registered in the second rainy season (March to May) and the main rainy season (June to September) with a magnitude of 1775 and 1903 cases, respectively. The number of COVID-19 cases declined following the main rainy season, from October to January. The decline in COVID-19 cases between October and January links to the lowest amount of rainfall, while the increased number of COVID-19 cases between March–May and June–September links to increased seasonal rainfall (Fig. 4b). The average temperature shows a decreasing trend during the peak outbreak period (Fig. 4a).

Taking into account the lag effects of climatic factors, wind and relative humidity increased prior to the day of detection of COVID-19 cases (Fig. 4c,d). The increased number of COVID-19 cases between March and September of the study period corresponded with decreased sunshine duration (Fig. 4e).

## Discussion

This study is the first to investigate the effect of climatic factors such as rainfall, temperature, humidity, wind speed and sunshine duration on the daily reported number of COVID-19 cases using different lag times (ranging from 0 to 14 days) in Addis Ababa, Ethiopia. We found some climatic factors, at lag periods prior to the detection of infection, had a significant effect on COVID-19 case frequency. Although study limitations need to be taken into consideration when interpreting these findings, it showed that there are opportunities for policymakers to incorporate climatic data in COVID-19 transmission management plans. Study limitations include the lack of data on the impact of other key climatic and non-climatic factors which might influence COVID-19 transmissions such as testing capacity, sanitization attitudes, public isolation policies, population density, population mobility, population immunity, ultraviolet light intensity, hospital admissions, air pollutant, and air quality index. The other limitation was that the study was conducted in Addis Ababa only and did not cover other parts of the country.

Our study found that a 1% increase in relative humidity was significantly associated with a 1.1% and 1.2% reduced COVID-19 caseload at 4 and 5 lag days prior to COVID-19 detection. This finding is consistent with a previous study conducted in the USA which found that the minimum and maximum significant effect of relative humidity on COVID-19 cases were observed between lag 3–6 days^23^. Similarly, our results are in agreement with other studies that show high humidity reduces COVID-19 transmission^17^. In contrast, a study conducted in 16 countries in Africa, demonstrated that a 1% increase in relative humidity reduced the COVID-19 incidence rate by 3.6% on the same day (lag 0)^11^.

Our study also showed that average temperature and sunshine duration were not significantly associated with the transmission of COVID-19 outbreaks, for the entire cumulative lag days during the study period in Addis Ababa. Studies in South Africa, Canada, Australia, and Pakistan also reported a non-significant association between temperature and COVID-19 incidence^14,24–26^. In contrast to these findings, studies conducted in India and Bangladesh found that increasing temperature was significantly associated with increasing number of COVID-19 cases^12,27^. The variations across studies may reflect the differences in the impacts of temperature on COVID-19 transmission between climatic zones.

A significant direct relationship between wind speed and the transmission of COVID-19 in Addis Ababa was also observed in our study. A 1 m/s increase in wind speed was associated with 1.8 times increase in COVID-19 confirmed cases. Wind speed is likely to increase the circulation of suspended respiratory droplets in the air and it may also influence the spread distance and diffusion rate of the virus. Similar findings that report a direct effect of wind speed on COVID-19 transmission were reported in other studies^27–31^. In contrast to these findings, an inverse relationship^13,32,33^ and a non-significant relationship^13^ between wind speed and COVID-19 transmission have been reported in previous studies.

The study rainfall analysis shows a 1 mm increase in rainfall is associated with 1.078 times increase in the daily number of COVID-19 cases, at a lag of 14 days. Our results are in agreement with studies undertaken in the USA that reported precipitation had a significant effect on COVID-19 cases until the rainfall reached 50.26 mm for all cumulative lag effects^23^. Another study undertaken in the USA found daily COVID-19 cases to increase with increasing rainfall between 32 and 44 mm but for case incidence to decrease over 44.9 mm^34^. However, the associational effect revealed that rainfall had a significant direct effect on COVID-19 cases for all lag periods except for the day of COVID-19 detection and 1 lag day.

With increased cumulative effects of rainfall and wind speed (prior to detection of COVID-19 cases), the number of positive COVID-19 cases significantly increased over the study area as compared with the rest of the variables included in the model. This means that when rainfall and wind speed are taken into consideration together there is an increased cumulative effect of COVID-19 cases. Our study results align with research undertaken in India that shows weather factors such as rain and wind increase COVID-19 transmission as lag days increase^35^. Although we did not have data to justify the scientific background regarding the impacts of climate factors on COVID-19, previous experimental studies showed that SARS-CoV-2 persistence on surfaces or in the air is sensitive to temperature, humidity, and ultraviolet light^6,7^. Environmentally sensitive respiratory viruses show seasonal transmission that coincides with changes in temperature, humidity, and solar radiation. Therefore, like other viruses with a lipid envelope, SARS-CoV-2 is probably sensitive to temperature, humidity, and solar radiation; this affects its ability to persist on surfaces and in the air and might have subtle impacts on transmission.

In our study, COVID-19 prevalence shows seasonal variation with the highest number of cases reported between June and September (i.e., during the main rainy season) and between March and May (i.e., during the second rainy season). The lowest number of COVID-19 cases was reported between October and January (i.e., during the dry seasons). The seasonal variations in COVID-19 cases coincide with the seasonal variation in climatic factors such as relative humidity, rainfall, and wind speed. The findings of our study show climatic factors have a significant effect on the transmission of COVID-19 in Addis Ababa. Intensive public awareness campaigns and governmental decisions that regulate the rate of mass gatherings are required during peak COVID-19 transmission seasons^36^.

### Policy implication

The findings of this study provided important information for policymakers to consider climatic factors when designing various preventive measures such as vaccination, social distancing and distributing personal protective equipment for the control and prevention of COVID-19 transmission in the community. For instance, the seasonal patterns of COVID-19 infections suggested that measures to limit the spread of COVID-19 infection should be targeted at specific times of the year with the highest transmission risk occurring for a maximum impact. Using climatic data, policymakers can also predict the future burden of COVID-19 and can identify a high-risk area for targeted interventions.

Although several studies have examined the transmission dynamics of the COVID-19 pandemic in different parts of the world^37,38^, evidence is still scarce and mostly limited to a few countries, particularly in Africa. Thus, our study provides additional information to understand the complex interactions between climatic factors and COVID-19 transmission in the Ethiopian Capital City (Addis Ababa), which reported the highest number of COVID-19 cases during the pandemic. Our results indicated that COVID-19 transmission was mostly related to three main climatic factors (humidity, rainfall, and wind speed) and COVID-19 cases have shown seasonal variations with the highest number of cases reported during the rainy season and the lowest number of cases reported during the dry season. The results of this study support some previous findings about the main climatic determinants of COVID-19 transmission^39,40^, which may be useful for decision-making and management of the disease.

## Conclusion

Our study found that climatic factors such as humidity, rainfall, and wind speed influence the transmission of COVID-19 in Addis Ababa. COVID-19 cases have shown seasonal variations with the highest number of cases reported during the rainy season and the lowest number of cases reported during the dry season. These findings may help the Ministry of Health to design strategies for the prevention and control of COVID-19 prior to the rainy seasons. Further nationwide studies are required to understand the association between climatic factors and COVID-19 transmission across all parts of the country.

## Data Availability

The raw COVID-19 case data used during this study are freely available from the repository hub of the National Data Management Center for Health at the Ethiopian Public Health Institute.
